# Identification of Voxels Confounded by Venous Signals Using Resting-State fMRI Functional Connectivity Graph Community Identification

**DOI:** 10.3389/fnins.2015.00472

**Published:** 2015-12-16

**Authors:** Klaudius Kalcher, Roland N. Boubela, Wolfgang Huf, Christian Našel, Ewald Moser

**Affiliations:** ^1^Center for Medical Physics and Biomedical Engineering, Medical University of ViennaVienna, Austria; ^2^MR Centre of Excellence, Medical University of ViennaVienna, Austria; ^3^Department of Radiology, Tulln Hospital, Karl Landsteiner University of Health SciencesTulln, Austria; ^4^Brain Behaviour Laboratory, Department of Psychiatry, University of Pennsylvania Medical CenterPhiladelphia, PA, USA

**Keywords:** fMRI, BOLD, graph analysis, graph clustering, physiological signals, brain, veins

## Abstract

Identifying venous voxels in fMRI datasets is important to increase the specificity of fMRI analyses to microvasculature in the vicinity of the neural processes triggering the BOLD response. This is, however, difficult to achieve in particular in typical studies where magnitude images of BOLD EPI are the only data available. In this study, voxelwise functional connectivity graphs were computed on minimally preprocessed low TR (333 ms) multiband resting-state fMRI data, using both high positive and negative correlations to define edges between nodes (voxels). A high correlation threshold for binarization ensures that most edges in the resulting sparse graph reflect the high coherence of signals in medium to large veins. Graph clustering based on the optimization of modularity was then employed to identify clusters of coherent voxels in this graph, and all clusters of 50 or more voxels were then interpreted as corresponding to medium to large veins. Indeed, a comparison with SWI reveals that 75.6±5.9% of voxels within these large clusters overlap with veins visible in the SWI image or lie outside the brain parenchyma. Some of the remaining differences between the two modalities can be explained by imperfect alignment or geometric distortions between the two images. Overall, the graph clustering based method for identifying venous voxels has a high specificity as well as the additional advantages of being computed in the same voxel grid as the fMRI dataset itself and not needing any additional data beyond what is usually acquired (and exported) in standard fMRI experiments.

## 1. Introduction

Any interpretation of fMRI results as indirect measures of neuronal activation rests on the assumption that magnetization changes caused by changes in blood oxygenation are due to brain activity in the immediate vicinity. Whether and to what extent this assumption holds, however, has been the matter of much debate from the first days of fMRI onwards. While the discussion of this “brain or vein” question has generated a wealth of research on identifying veins and signals originating from them, the majority of fMRI studies still ignore the issue and take no measures to assess or reduce the influence of signals from major vessels (Menon, 2012).

Quantification of the influence of draining veins on fMRI results has provided evidence that it is most pronounced at low magnetic field strengths, and the relative influence of microvasculature to the MR signal increases as the field strength increases (Duong et al., 2003): while at 1.5 T the signal originates virtually entirely in the macrovasculature (Lai et al., 1993), the ratio shifts in favor of the microvasculature as a major signal source at 3T, 4T, and 7T. Still, even at higher field strengths, protocols using gradient echo EPI are highly sensitive to signal changes originating from larger veins, to the point that no significant improvement can be observed between 3T and 7T results (Geißler et al., 2013).

Several efforts have been made to reduce the influence of venous signals on fMRI measurements, and a number of effective ways for increasing the specificity of signals for the microvasculature have emerged from them. The use of spin-echo instead of gradient echo sequences can drastically reduce extravascular signal contributions (Duong et al., 2003), but only at a steep cost in terms of signal-to-noise and contrast-to-noise ratio (Norris, 2012). Specific corrections to eliminate venous signals based on phase images have also been developed (Menon, 2002; Rowe and Logan, 2005; Curtis et al., 2014), but are rarely included in the sequences used by typical fMRI studies, and yield the risk of introducing errors through over-correction (Nencka and Rowe, 2007).

The development of these methods for identifying veins is to some extent related to that of methods for eliminating physiological influences, leading to converging developments. One of the most common approaches to address physiological signal contamination is RETROICOR (Glover et al., 2000), which uses externally measured respiration and cardiac signals for a regression-based correction of fMRI data. A later method termed CompCor (Behzadi et al., 2007) eliminates the need for externally measured signals by identifying potential regressors from either ventricular and white matter signals, or from signals in voxels with higher-than-average time course standard deviation—a feature typically seen in voxels containing larger veins. The most recent development, Highcor, merges this line of research with the work on phase-based venous suppression mentioned above by using the correlation between phase and magnitude image time series to identify venous voxels that can be used to extract regressors for physiological noise reduction (Curtis and Menon, 2014).

The reasons for identifying venous voxels are more complex than the elimination of global physiological noise, however, as any BOLD effects necessarily carry the potential for signal changes further downstream along draining veins. It is thus desirable to not only reduce global physiological noise as in these regression approaches, but also to reliably identify voxels with potential venous signal contributions to help interpreting signal changes seen in and around them. The most direct way of localizing macrovascular effects might be the creation of venous maps using susceptibility weighted imaging (SWI; Reichenbach et al., 2000; Haacke et al., 2004). Still, extravascular signal influences in fMRI measurements might extend beyond the delineation of veins in the SWI image, and imperfect coregistration of SWI to fMRI images can further limit the precision of this method of localization. An immediate measure proposed to minimize macrovascular influences is the elimination of voxels with time series coefficients of variation much larger than the local average of their surrounding voxels [a concept related to the second of the two approaches used by Behzadi et al. (2007) in CompCor, see above], which empirically corresponds to regions next to large vessels, as done in the minimal preprocessing pipeline (Glasser et al., 2013) of the Human Connectome Project (Van Essen et al., 2013). This method has the advantages of requiring neither separate protocols nor specialized measurement techniques, and estimates affected voxels directly from standard fMRI time series; however, there exists no evaluation of the correspondence of the time series standard deviation with venous effects.

The question of which voxels are influenced by large vessels is not a purely theoretical exercise. While in the beginnings of fMRI, the brain-vs.-vein debate was settled by a general acknowledgment that with the imaging resolutions then available, the practical relevance of knowing whether a signal originates in the microvasculature in the cortex or in the draining vein at its surface was limited. There are multiple reasons why this argument should no longer be used as an excuse for avoiding the question. First, many veins run in sulci and might cause a signal change whose causal origin is on the gyrus on one side of the sulcus be misattributed to the gyrus on the other side. While earlier imaging techniques might not have allowed to make such distinctions regardless of whether the measured signal originated in the parenchyma or in a draining vein, many current analysis techniques like surface projections for surface-based analyses rely on correct attribution of signals at this level. The second, somewhat related, reason is that improvements in spatial resolution at higher field strengths and with the use of more sophisticated acceleration techniques (Simultaneous Image Refocused EPI, Multiband EPI) have led to the possibility of imaging at sub-millimeter resolutions (Feinberg et al., 2010), but this improvement in nominal spatial image resolution can only lead to interpretable gains if the physiological basis for the measured effect matches this granularity. Indeed, Turner (2002) suggested that due to dilution effects, draining vein effects might not be seen at more than a few millimeters distance from the gray matter region drained where the effect originated. But while a spatial gap between neuronal origin and the immediate source of the measured BOLD signal of 4 mm might be considered of limited relevance when imaging at a spatial resolution of 3 mm, the existence of unavoidable spatial discrepancies of this magnitude would render advances into higher resolutions entirely pointless. Finally, it is possible to show that at least in some cases, draining vein effects might occur at much larger distances from their neuronal origins, as is the case for signal changes in the basal vein of Rosenthal (BVR) next to the amygdala (Boubela et al., 2015).

In the analysis of the BVR signals, it also became apparent that one distinguishing feature of venous voxels was their resting-state functional connectivity pattern (Boubela et al., 2015), exhibiting very strong positive and negative correlations to other voxels in the macrovasculature. Thus, in an approach similar to functional parcellation methods of the brain (Eickhoff et al., 2015) this resting-state connectivity structure between voxels could be used to distinguish voxels in the macrovasculature from others. One such approach consists in using graph learning tools on the connectivity graph (where vertices correspond to voxels or sets of voxels, and edges link vertices with correlated time courses together). Previously, connectivity analyses have rarely been performed on a voxel-wise level, among others for computational reasons: if, for example, 150,000 voxels lie within the brain mask, the complete voxel-by-voxel correlation matrix would consist of 2.25·10^10^ entries, taking up about 167 GB (in practice, the number of voxels is typically reduced by restricting analysis to gray matter voxels or by resampling the data to a coarser resolution). Not all of these entries actually need to be stored to perform analyses, in particular when analyzing a relatively sparse connectivity graph, and efficient tools for tackling similar problems on large datasets have been developed in other fields.

In this work, graph based cluster analysis was performed to show how these tools can be applied to solve a practical problem of fMRI data analysis. Voxel-by-voxel correlations are computed for all in-brain voxels to create a voxelwise connectivity graph. Resampling to a coarser resolution as well as limiting analysis to a subset of voxels (e.g., gray matter voxels) are avoided as they would hamper the specific research question: resampling would lead to a loss of specificity in that affected voxels would be averaged with adjacent voxels to form the larger voxels of the coarser grid, and a gray matter mask might exclude parts of the venous structure, effectively hindering the identification of venous voxels based on their connectivity to voxels within other veins. Based on previous observations (Boubela et al., 2015), the largest clusters with the strongest (positive and negative) correlations among their voxel's time-series emerging from a clustering of this graph could be expected to reflect medium to large veins.

## 2. Materials and methods

### 2.1. Subjects

Fifteen healthy subjects (8 females/7 males, mean age 35.3, SD 13.3) were recruited at Medical University of Vienna. Exclusion criteria were prior psychiatric or neurologic illnesses, as well as the usual exclusion criteria for MR studies. All subjects gave written informed consent prior to the scan and the study was approved by the local institutional review board (Ethikkommission der Medizinischen Universität Wien).

### 2.2. Data acquisition protocols

All MRI scans were performed on a 3 Tesla TIM Trio using the standard 32-channel head coil and whole-body gradients (Siemens Medical Solutions, Erlangen, Germany). First, a high-resolution anatomical image was acquired using MPRAGE with 1 × 1 × 1.1 mm^3^ resolution, and 160 sagittal slices (TE = 4.21 ms, TR = 2300 ms, flip angle 90°, inversion time 900 ms). Second, BOLD fluctuations at rest were measured with a short-TR multi-band EPI-sequence (Feinberg et al., 2010) using 1.7 × 1.7 × 2 mm^3^ resolution, 2 mm slice gap (matrix size 128 × 128, 32 axial slices, TE = 31 ms, TR = 333 ms, flip angle 30°, multiband factor 8, bandwith = 1776 Hz/Pixel) collecting 1200 volumes. Finally, susceptibility weighted images (SWI) were acquired at 0.6 × 0.6 × 2.0 mm resolution (matrix size 384 × 384, 52 slices per slab, 1 slab, TE = 29 ms, TR = 42 ms, flip angle 15°) to visualize medium to large venous vessels.

### 2.3. Preprocessing

To keep closely to the original images, only minimal preprocessing was applied to functional data, including only skull stripping using FSL BET, motion removal using FSL MCFLIRT, and band-pass filtering. For the latter, the pass-band used was 0.01–0.2 Hz, to avoid as far as possible influence from high-frequency respiratory or cardiac fluctuations. SWI images were segmented using FSL FAST for vein delineation, coregistered to the EPI weighted images, with the vein masks generated from segmentation also being transformed into EPI space using the resulting transformation parameters, using trilinear interpolation to ensure that all voxels in EPI space with some overlap with veins from the SWI mask have non-zero values. This vein map in EPI space was then binarized to generate a vein mask for the EPI images.

### 2.4. Graph generation

Pairwise Pearson correlation coefficients were computed between all voxels within the brain mask, using GPUs for the calculation of the correlation coefficients (Boubela et al., under revision) and splitting the dataset into tiles to allow for the computations to fit within GPU memory (6 GB). For each subject, this correlation matrix was thresholded to generate the adjacency matrix of a graph using a correlation threshold such that *S* < 4, with

$$\begin{array}{l} S=\frac{\log E}{\log K} \end{array}$$

(where *E* is the number of edges and *K* the average node degree). This thresholding criterion results in a rather sparse graph of only the strongest correlations, which are more likely to reflect adjacent voxels along the same vein or otherwise highly congruent voxel signals (as opposed to the more subtle long-distance connections of brain networks of neuronal origin; see below in the discussion for more details on the effect of the sparsity criterion). For each subject, the largest correlation threshold fulfilling *S* < 4 was computed iteratively by decreasing the threshold in steps of 0.01, starting at 1. The threshold was applied to the absolute value of the correlation coefficients to take into account both positive and negative correlations exceeding a certain correlation strength. The resulting connectivity graph had all in-brain voxels as vertices, and each correlation between two voxels that was above the threshold resulted in edges between the two corresponding vertices, with the correlation coefficient used as edge weight. Graphs were represented using the package igraph (version 1.0.1) in R (version 3.1.1). Self-loops and multiple edges were eliminated using the igraph function simplify.

### 2.5. Graph cluster identification

Community identification on the graph was performed using the method based on modularity optimization by Newman (2006) as implemented in the igraph function cluster_fast_greedy. The optimization of graph modularity means that the resulting clusters are defined by their voxels having maximum connectedness among each other and minimum connectedness to voxels outside their own cluster. All voxels from all clusters that individually contained 50 or more voxels were then pooled into a single mask, which thus contained all voxels with time-courses strongly correlated (either positively or negatively) with those of a large number of different voxels. It should be noted that Newman's method is intended to detect communities in connected networks and that its application on sparse networks as used here might result in some cases in entire connected components being categorized as clusters. Nonetheless, for our purposes, this is still sufficient to detect groups of voxels with highest relative connectedness to each other considering the general sparsity of the graph.

### 2.6. Validation

To show that these voxels correspond mostly to vasculature, the overlap with the vein mask from SWI was computed (in EPI space); the proportion of voxels from the graph clustering map that overlaps with the SWI vein mask can be seen as a measure for the specificity of the graph clustering method, though it should be kept in mind that it is not the true specificity because the segmented SWI is not the ground truth for the identification of venous voxels: coregistration imperfections can lead to spatial deviations in the localization of these voxels, and not all low signal intensities in SWI originate from veins since other factors like iron levels (higher in the basal ganglia than in the rest of the brain) or proximity to air cavities or bone affect susceptibility. The latter observation also implies that it is impossible to make any meaningful quantification of the sensitivity of the graph clustering method by using SWI, as it means that an accurate map of venous voxels should not indiscriminately include all voxels with low signal intensity in SWI.

## 3. Results

Overall, of the voxels within the brain masks (between 142,800 and 172,300 for the different subjects, mean 157,600, SD 10,540), 17,730 ± 5069 voxels (or 11.2 ± 3.0%) were identified by the graph clustering algorithm as being part of large highly coherent networks (see Table 1).

**Table 1:** **Quantitative overview of the voxels identified by graph clustering, and comparison to segmentation of SWI**.

	**Minimum**	**1st Quartile**	**Median**	**Mean**	**3rd Quartile**	**Maximum**
Correlation threshold	0.63	0.72	0.75	0.79	0.88	0.95
Voxels in brain mask	142,800	148,900	154,400	157,600	167,100	172,300
Voxels in clustering mask	10,470	13,600	18,400	17,730	20,940	26,760
Idem, in % of brain mask	6.5%	9.4%	11.1%	11.3%	12.4%	17.9%
Overlap with SWI veins	58.8%	63.7%	68.4%	67.1%	70.8%	72.5 %
Overlap with SWI veins or brain edge	64.5%	72.9%	78.3%	76.6%	79.7 %	87.6%

*The minimum, first quartile, median, mean, third quartile, and maximum across all subjects is given for the correlation threshold obtained for the binarization of the correlation matrix to a graph adjacency matrix, the absolute numbers of voxels within the brain mask and within the graph clustering mask, the percentage of voxels of the brain mask that were within the clustering mask, as well as for the percentage of voxels of the graph clustering map that lie within the mask generated from SWI segmentation (which can be interpreted as a measure of specificity), without and with the additional inclusion of brain edge voxels*.

**Figure 1:**
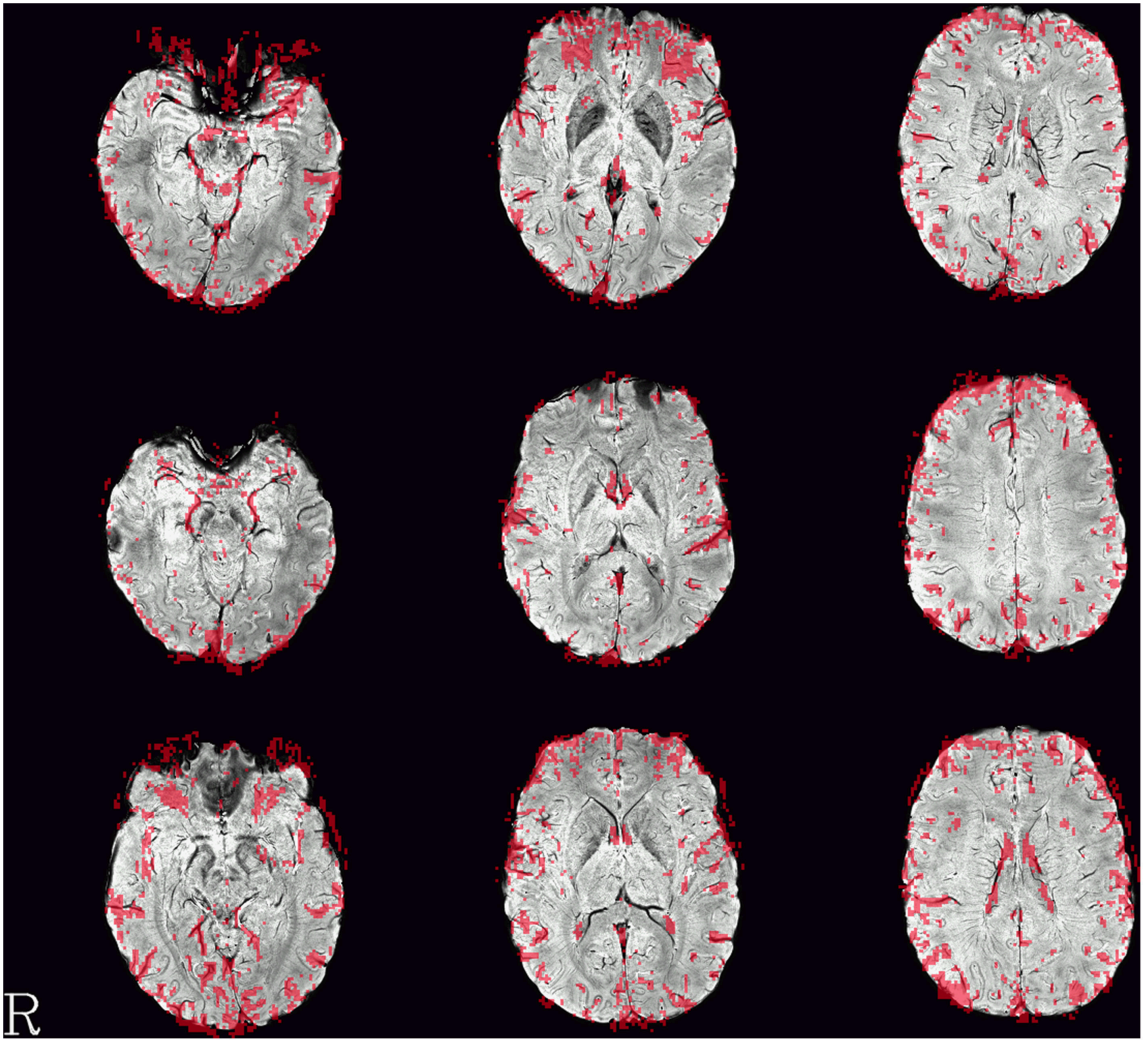
**Example single-subject graph clustering masks overlaid on the respective subject's SWI**. Red areas indicate voxels within the venous voxel mask gained from graph clustering, each row showing data from a different subject. Structures seen in the graph clustering maps tend to reflect veins seen in SWI. Note that the delineation of those veins in SWI and the voxels in the EPI influenced by the signal as identified from the BOLD EPI itself can deviate slightly. Slices were selected to contain larger veins running along the slice orientation in the SWI.

Single-subject images of the graph clustering masks overlaid over SWI are shown in Figure 1. The spatial distribution of the voxels identified by the method seems to exhibit a consistent pattern. Most of the voxels within the brain follow the path of veins visible in the SWI underlay. Another set of voxels delinates areas of low signal quality in orbitofrontal regions subject to susceptibility artifacts or at the edge of the brain, in either case such voxels could be discarded for fMRI analyses interested in neuronal effects.

**Figure 2:**
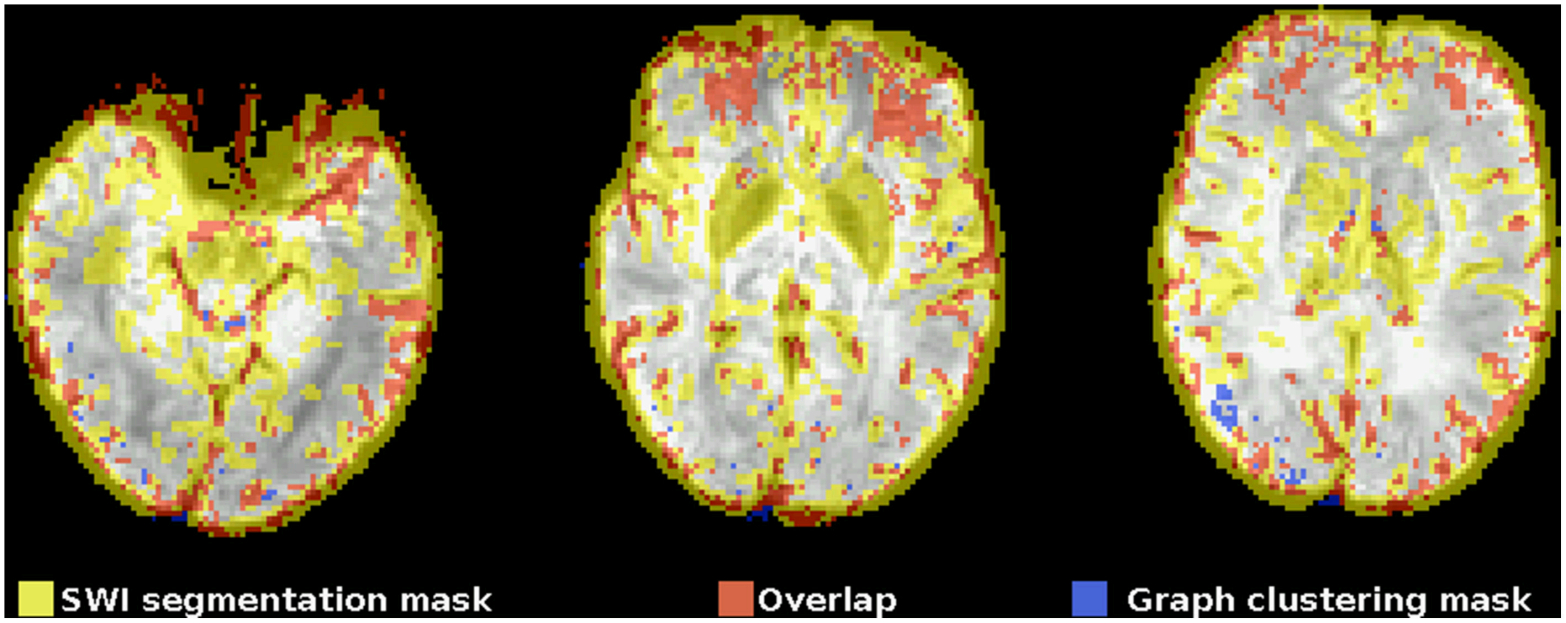
**Example comparison between the segmented SWI and graph clustering mask of one subject (the subject shown in the top row in Figure 1)**. The underlay is the SWI, coregistered, and resampled to the EPI space. Voxels within the mask gained from the segmented SWI are yellow, voxels from the graph clustering mask that are within the SWI mask are red, voxels from the graph clustering mask that do not overlap with the SWI mask are blue. Note how the SWI segmentation mask tends to be rather unspecific to veins in regions with susceptibility-related low signal intensities of different origin, most notably in the basal ganglia due to their high iron levels.

This observation can also be quantified by comparing the mask gained from graph clustering with a binarized mask gained from segmenting the SWI image (see Figure 2), and the average proportion of voxels of the mask identified via graph clustering overlapping with veins in SWI is 0.67±0.05. A further significant proportion of voxels not directly overlapping veins lies on the edge of the brain mask, as identified by eroding the brain mask with a 5 × 5 × 5-voxel kernel (see Figure 4) using the R package mmand (Clayden, 2014), outside of what can be recognized as the brain itself (BET seems to be rather conservative in skullstripping), possibly reflecting signals from superficial veins, raising the overlap proportion to 0.77 ± 0.06 (see also Table 1).

In some brain areas (e.g., in the medial prefrontal region in Figure 1), the locations of the vein recognizable in SWI on one hand and the voxels of the graph clustering mask on the other can be observed not to overlap perfectly. Still, the similarity of the shape between the two features, only shifted by 1–2 voxels, strongly suggests that they are caused by the same underlying structure (i.e., the same vein). Such discrepancies are not necessarily worrying. Since the graph clustering mask is generated from the EPI voxel timecourses themselves as opposed to the SWI images acquired in a separate measurement, they can be seen as yielding potentially valuable complementary information on the effect of a vein on the EPI measurement.

**Figure 3:**
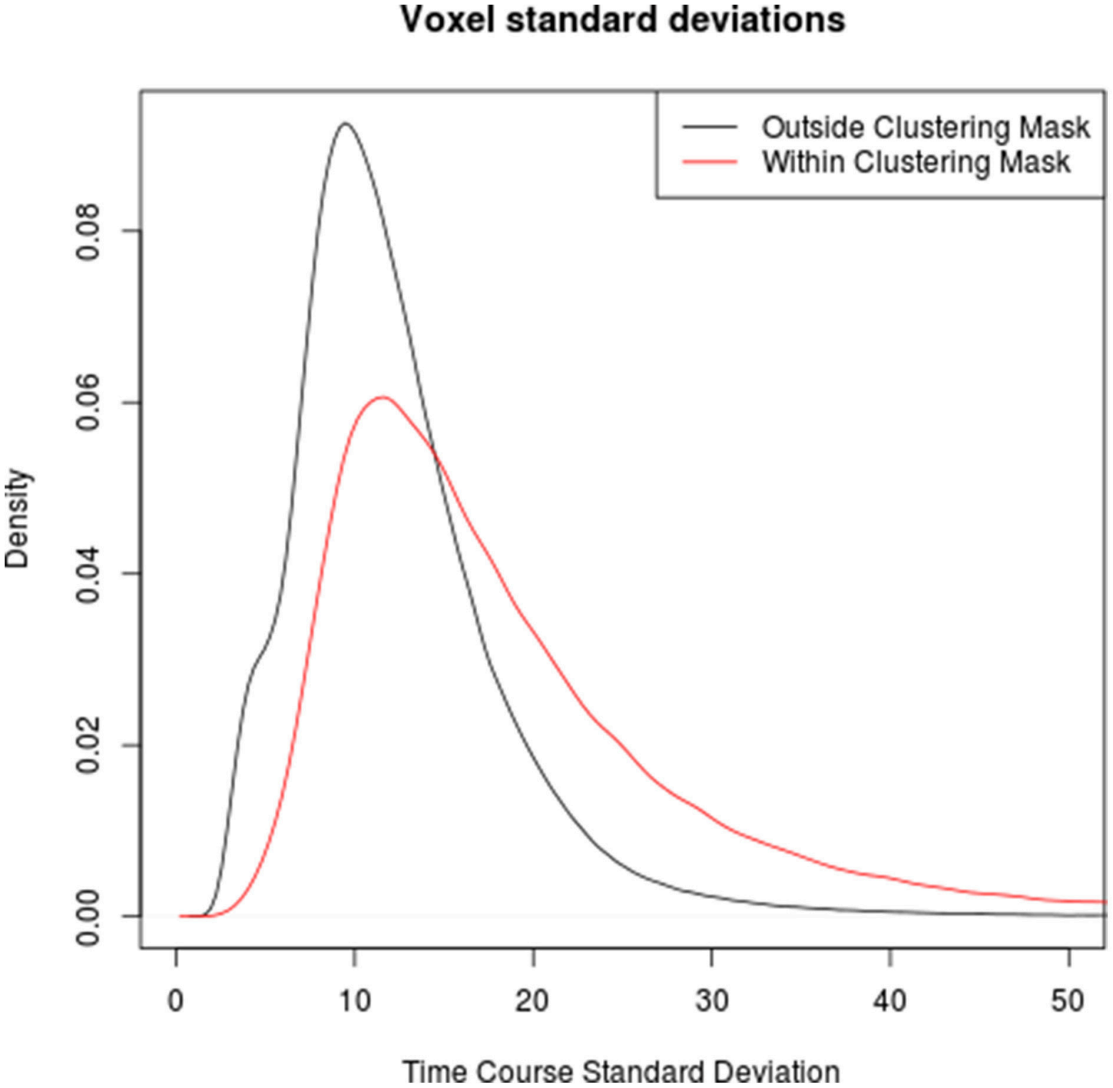
**Comparison between the distributions of time series standard deviation in voxels outside (black) and within (red) the clustering mask over all subjects**. Note that while time series standard deviations in voxels identified as veins tends to be higher than in those outside veins, the boudary is not clear-cut, highlighting the limits of venous voxel identification based on time series standard deviations.

Comparing the time series standard deviations of voxels within and outside the clustering brain mask reveals that voxels within the mask indeed have on average significantly higher standard deviations (*p* < 2·10^−16^ in all individual subjects), in congruence with the underlying assumption of the method used by Glasser et al. (2013). However, the overlap between the signal standard deviations in macro- and microvasculature is very pronounced in all subjects, suggesting that a threshold based on the signal standard deviation alone might not be sufficient to discriminate between the two types of voxels (see Figure 3).

**Figure 4:**
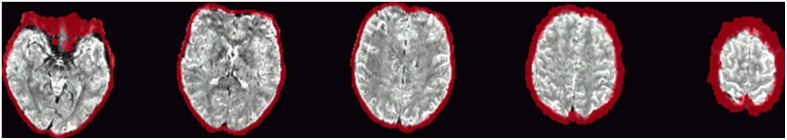
**Example of the definition of “brain edge” voxels, marked in red, and defined by erosion of the brain mask by a 5 × 5 × 5-voxel kernel**.

## 4. Discussion

The results presented here show that graph based brain network analysis on a voxelwise basis can yield important insights on the origins of the underlying signals. In particular, network clustering yields a set of voxels defined by strong connections among each other and weak connectivity to voxels outside of this set that contains mostly voxels in medium to large veins (as identified by SWI) as well as extra-cerebral voxels. The very structure of these clusters, voxels spread across the whole brain with a large number of connections with correlation strengths above 0.7–0.95 (depending on the subject), strongly suggests that these voxels contain little information related to local neuronal activity, and rather reflect blood oxygenation changes on a larger (physiologic) scale. These voxels thus violate the underlying assumption of most fMRI analyses that BOLD signal changes in a voxel can be interpreted as an indirect measure of local neuronal activations, and should thus be excluded in this type of analysis.

It is worth noting that some correlation patterns typically arising in fMRI datasets are not reflected in the results computed here and do not seem to confound the correlation-based vein identification. The first of those is that in task fMRI, large vessels often correlate with task signals in active brain regions as they are draining the blood from those regions. One might thus expect that conversely, there should be a correlation between the venous signals investigated here and the signals from voxels within the parenchyma that are drained by these veins, making it difficult to distinguish venous voxels from parenchyma voxels. This does not seem to occur here, as indicated by the overlap of most voxels in the clustering mask with venous voxels identified by SWI. One reason for this might be that the correlation strength between the signals within the vein and each of the individual regions drained by the vein is substantially below the relatively high correlation thresholds that emerge from the high S threshold used in network creation. For medium to large veins, each gray matter voxel ultimately drained by them contributes only a small part to the signal in voxels within the vein, thus leading to lower correlation strengths between parenchyma voxels and veins of this scale in accordance with theoretical models of downstream dilution of effects in veins. This might explain why the clustering mask includes only larger vessels, and fails to identify some of the smaller vessels appearing in the SWI image. The reason for the absence of the correlations between signals from large veins and large areas of activation that typically occur in task fMRI might be that in task fMRI, there is an artificially high coherence of a particular (set of) brain region(s) with the signal in the vein due to the task-induced structure in these activations. In resting-state data as used in this study, however, the patterns along which all regions draining into a particular vein contribute to its signal are less coherent among each other, with different regions potentially contributing differently to the venous signal, and thus having lower individual correlations with it.

**Figure 5:**
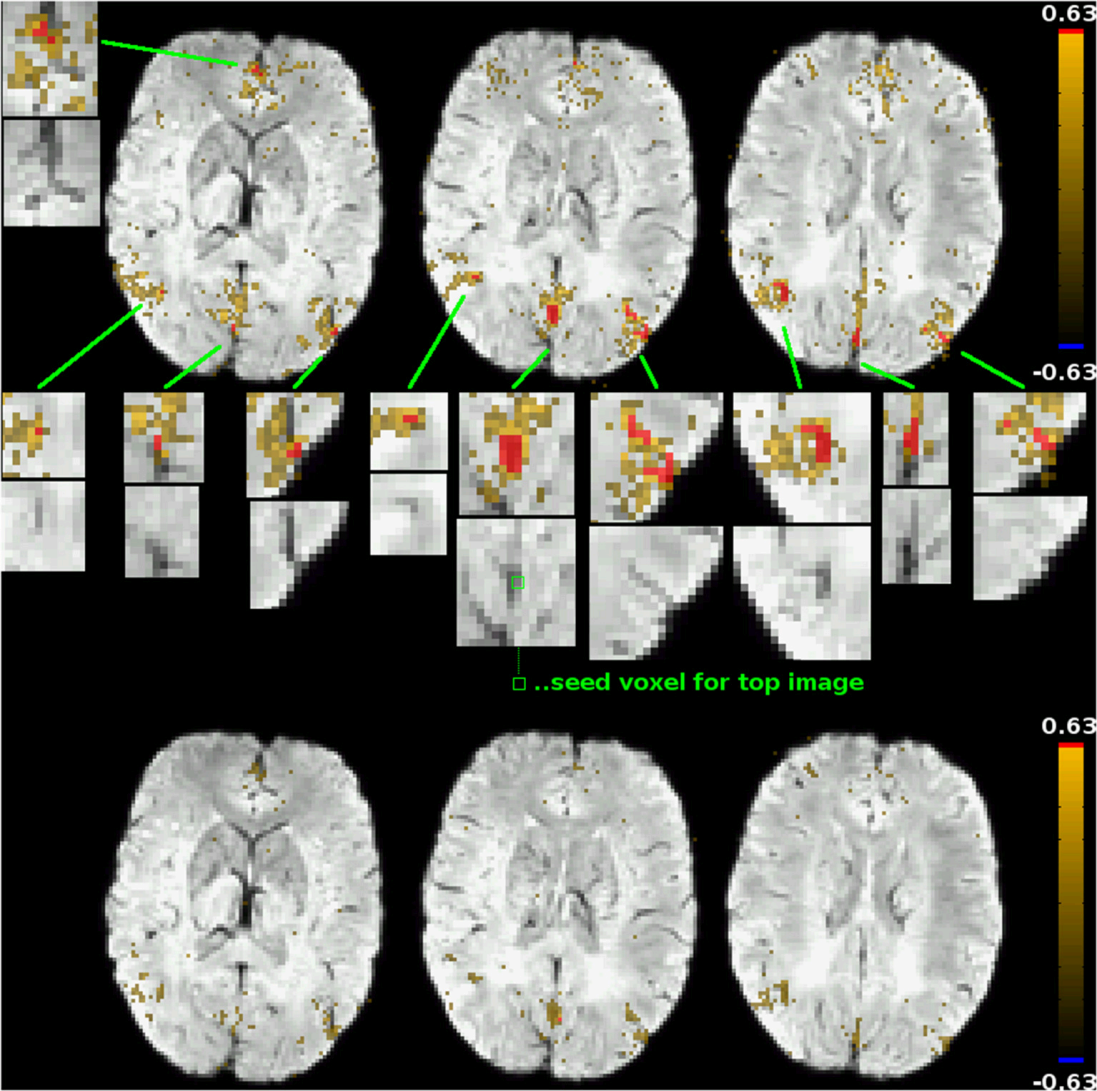
**Functional connectivity maps for two adjacent voxels in the PCC: one within a vein as identified by SWI (top subfigure; seed location marked out in the central zoomed insert), one outside of large veins (bottom subfigure; seed location identifiable as a red dot in the connectivity map)**. The underlay is the subject's SWI, coregistered to EPI space. Voxels exceeding that subject's correlation threshold for graph binarization (0.63) are marked out in red, maps are thresholded at a correlation coefficient of 0.25. Only for the venous seed do correlations exceeding this threshold exist; they are shown in zoomed inserts, along with the corresponding picture detail from the underlay alone. All voxels with a correlation coefficient exceeding the binarization threshold can be attributed to veins identifiable in the underlay. Correlations to other voxels of the default-mode network, including non-venous voxels, can be seen for both seeds, but correlation strengths in these voxels do not exceed the binarization threshold.

The second type of correlation pattern that might be expected to be visible in a functional connectivity graph are resting-state networks previously described in the literature, such as the default-mode network (DMN) or the left and right fronto-parietal networks. The reason for them not appearing in the cluster mask is that in unblurred datasets as those used in this study to compute the correlation graphs, the correlations between parenchyma voxels in these networks are much lower than those between venous voxels, and the high correlation threshold used to construct the graphs ensures that only the latter are reflected in it. This subtlety of voxel time course correlation patterns is easily lost when using only blurred datasets, but can be visualized effectively using the DMN as an example. Figure 5 shows the functional connectivity of two voxels in the posterior cingulate cortex (PCC), a main constituent of the DMN, one of them a voxel clearly in a vein as identified by SWI (its functional connectivity map being shown in the top part of the figure), the other being an adjacent voxel outside the vein (its functional connectivity map is shown in the bottom part of the figure). For the venous voxel, the correlation coefficients in adjacent venous voxels exceed 0.63, which was the threshold for graph construction in that particular subject (based on the network sparcity criterion of *S* = 4; this was the lowest connectivity treshold for all subjects, see Table 1), as well as a number of other voxels in typical DMN regions in the medial prefrontal cortex as well as bilaterally in the parietal cortices. On closer inspection, one notices that all of those voxels can be related to veins identifiable on the SWI image (see zoomed inserts). Correlations with other voxels in DMN regions, including voxels further from large visible veins, also exist, but with far lower correlation strengths. When using a seed outside of veins, as exemplified by the connectivity map in the lower part of the figure of an adjacent voxel in the parenchyma, correlations above the threshold can be found neither in other DMN regions nor even in adjacent voxels. Indeed, the highest correlation coefficient found anywhere in the brain for that particular seed is 0.49—far away from the threshold of 0.63. This is consistent with results typically obtained from blurred resting-state datasets: signal time courses from veins draining one part of a resting-state network can be seen as reflecting the signal time courses of the voxels in the gray matter that these veins drain in a way similar to how the time course of a voxel in a blurred dataset reflects the time courses of the voxels in its neighborhood, and time courses of voxels in the draining veins of different parts of a resting-state network are more strongly correlated with each other in the same way as correlation strengths between voxels in different parts of a resting-state network are increased in spatially blurred datasets. Correlation strengths between parenchyma voxels of resting-state networks are lower, and thus, if the correlation threshold used to generate a binarized graph from the voxelwise correlation matrix is high enough, these parenchyma voxel correlations are not reflected in the graph analyses performed after binarization, and only the connections between venous voxels remain in the graph and, ultimately, define the graph modules.

Of course, this work is not the first attempt at identifying artifactual signals in fMRI datasets. Previous attempts include both the use of complementary measurements (including SWI) as well as the localization of its effects based on EPI measurements alone. The advantage of using EPI-based identification over complementary measurements is the more immediate relationship to the application of the results to the fMRI analyses in question. In the results presented here, this can be seen in the slight differences in localization of venous voxels between the clustering and SWI-based masks in the context of general correspondence between the two (see for example Figure 1). While the general similarity between the features seen in the SWI and clustering masks corroborates the theory of venous origins of the signals seen in the graph cluster voxels, the difference in location highlights that imperfect correspondence between two different modalities, emerging from imperfect coregistration, geometric distortion or other origins, necessarily limit the use of inference from complementary measurements for the identification of affected voxels in the EPI image. In this sense, vein identification methods using EPI and SWI complement each other as different, independent modalities, and both are important to provide a link between the results from EPI images to signal sources not directly visible in BOLD EPI such as most veins. While SWI yields the more anatomically accurate maps of the venous architecture in a subjects' brain, methods based on post-processing techniques applied on the BOLD EPI data themselves add to this a more direct view on the immediate effect of these veins on fMRI measurements.

Among methods based on EPI measurements, two categories can be distinguished, the first being methods using externally measured signals and correlating them with time courses from the EPI measurement, and the second being methods based on the analysis of EPI time courses by themselves. The first category typically uses high-frequency physiological nuisance signals (usually heart rate and respiration monitoring), which, however, has a different spatial distribution than the venous signals investigated here (Windischberger et al., 2002): high-frequency physiological noise tends to be concentrated near arteries and the CSF, which is subject to the same pulsations, while the low-frequency physiological signals investigated here tend to be localized in or near the venous macrovasculature. Physiological low-frequency signals are acquired and analyzed only in very few studies, but studies using them have shown them to be quite useful in identifying blood flow related phenomena in fMRI datasets (Tong and Frederick, 2012; Tong et al., 2014). The use of peripheral measurements, however, has one practical and one more fundamental limitation. The practical limitation is the necessity of additional hardware and measurement overhead for their acquisition leading to such measurements not always being available for all fMRI datasets, and the potential for additional error sources in their acquisition. While this issue can be overcome in any given study if the necessary steps are taken prospectively, it cannot be employed when analyzing datasets acquired without measuring these peripheral physiological signals, as is often the case in investigations using data shared by other researchers (Biswal et al., 2010; Kalcher et al., 2012). A more fundamental issue, though, is the time delay involved between the recording of the physiological signals at the external measurement location (e.g., the fingertip or toe for pulse oxymetry) and the brain, or, to be more precise, different locations in the brain. With standard EPI sequences as currently used, with a TR of between 2 and 3 s, the effect of this issue is rather limited, but with the current development toward short-TR multiband EPI sequences with higher temporal resolutions, the difficulties arising from the delay between the peripheral acquisition of physiological signals and their effect in the brain become more pronounced.

Finally, the use of measures directly derived from the EPI time series has been mostly confined to computationally less complex methods (e.g., the voxelwise time course standard deviation, a variant of which has been used in the Human Connectome Project), in part due to the lack of tools for tackling the computational challenges posed by more sophisticated methods like the voxel-by-voxel graph clustering approach presented here. Readily available tools from the domain of big data analysis can be applied to overcome computational obstacles and open the way to more comprehensive analysis tools. The comparison of time course standard deviations within and outside the graph clustering mask (see Figure 3) confirms the rationale behind the Human Connectome Project's preprocessing step of eliminating voxels with higher than normal standard deviations, but at the same time suggests that a one-dimensional measure not taking into account the connection structure between voxels might not yield a clear-cut discrimination threshold, as values of this score for normal brain tissue voxels with relatively high signal standard deviation and venous voxels with relatively low signal standard deviations overlap substantially.

In contrast, voxelwise graph analysis can be a useful tool to identify voxels in the macrovasculature by their highly correlated low-frequency signals. This latter point should be highlighted, as the band-pass filter applied (0.01–0.2 Hz) eliminates the possibility that the correlated signals in those voxels can merely be attributed to large-scale physiological noise (e.g., of respiratory or cardiac origin), which would have a higher frequency signal spectrum. Instead, they might exhibit more problematic signal fluctuations in the low-frequency domain, easily misattributed to local low-frequency fluctuations. In addition, the presence of such fluctuations might also be indicative of a risk of seeing downstream activations due to venous drainage of activations at more distant voxels, as it occurs during some emotional-visual tasks in the BVR (Boubela et al., 2015). The identification of voxels at risk is thus a powerful tool to increase specificity in the interpretation of fMRI BOLD activations.

### Conflict of interest statement

The authors declare that the research was conducted in the absence of any commercial or financial relationships that could be construed as a potential conflict of interest.
